# Multicenter randomized phase II study comparing docetaxel plus curcumin versus docetaxel plus placebo in first‐line treatment of metastatic castration‐resistant prostate cancer

**DOI:** 10.1002/cam4.3806

**Published:** 2021-03-05

**Authors:** Judith Passildas‐Jahanmohan, Jean‐Christophe Eymard, Mélanie Pouget, Fabrice Kwiatkowski, Isabelle Van Praagh, Laurent Savareux, Marc Atger, Xavier Durando, Catherine Abrial, Damien Richard, Angeline Ginzac Couvé, Emilie Thivat, Brigitte Monange, Philippe Chollet, Hakim Mahammedi

**Affiliations:** ^1^ Centre Jean Perrin Clermont‐Ferrand France; ^2^ Université Clermont Auvergne Centre Jean Perrin INSERM U1240 Imagerie Moléculaire et Stratégies Théranostiques Clermont‐Ferrand France; ^3^ Division de Recherche Clinique Délégation Recherche Clinique et Innovation Centre Jean Perrin Clermont‐Ferrand France; ^4^ Centre d'Investigation Clinique UMR501 Clermont‐Ferrand France; ^5^ Medical Oncology Department Institut Jean Godinot Reims France; ^6^ CHU de Clermont‐Ferrand Clermont‐Ferrand France; ^7^ Centre d'Urologie Auvergne Clinique de la Chataigneraie Beaumont France; ^8^ Service de Pharmacologie Médicale UMR Inserm 1107 Neuro‐Dol Centre Hospitalier Universitaire Université Clermont Auvergne Clermont‐Ferrand France; ^9^ Medical Department Emile Roux Hospital Puy‐en‐Velay France

**Keywords:** chemotherapy, curcumin, docetaxel, metastatic castration‐resistant prostate cancer, phase II, randomized trial

## Abstract

**Background:**

Metastatic castration‐resistant prostate cancer (mCRPC) patients have a poor prognosis, and curcumin is known to have antineoplastic properties. On the basis of previous phase I and phase II studies, we investigated whether the association of curcumin with docetaxel could improve prognosis among mCRPC patients.

**Methods:**

A total of 50 mCRPC patients (included from June 2014 to July 2016) treated with docetaxel in association with oral curcumin (6 g/d for 7 days every 3 weeks) versus placebo were included in this double‐blind, randomized, phase II study. The primary endpoint was to evaluate the time to progression. Among the secondary endpoints, compliance, overall survival, prostate‐specific antigen (PSA) response, safety, curcumin absorption, and quality of life were investigated. An interim analysis was planned in the modified intention‐to‐treat population with data at 6 months (22 patients per arm).

**Results:**

Despite good compliance and a verified absorption of curcumin, no difference was shown for our primary endpoint: progression‐free survival (PFS) between the placebo and curcumin groups was, respectively, 5.3 months versus 3.7 months, *p* = 0.75. Similarly, no difference was observed for the secondary objectives: PSA response rate (*p* = 0.88), overall survival (*p* = 0.50), and quality of life (*p* = 0.49 and *p* = 0.47).

**Conclusion:**

Even though our previous studies and data in the literature seemed to support an association between curcumin and cancer therapies in order to improve patient outcome and prognosis, the results from this interim analysis clearly showed that adding curcumin to mCRPC patients’ treatment strategies was not efficacious. The study was discontinued on the grounds of futility.

## Graphical abstract

In this manuscript, we present the result of the interim analysis of our study conducted in metastatic castration‐resistant prostate cancer (mCRPC) patients. According to previous studies, we investigated whether the association of curcumin to docetaxel could improve prognosis among patients. This study is the first one to explore the efficacy of curcumin in mCRPC treated with docetaxel, with the assessment of serum curcumin levels. The results from this phase II study with such a posology lead us to conclude that curcumin in association with docetaxel does not improve mCRPC patients, nor does it improve progression‐free survival or overall survival.

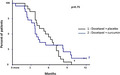

## 1 INTRODUCTION

Prostate cancer is the most common cancer among men in western countries (United States and western Europe) and the second most common worldwide.^1^ Androgen deprivation therapy (ADT) by hormonal or surgical castration remains the standard care for hormone‐sensitive prostate cancer.^2^ However, resistance to castration can occur and leads to metastatic progression in most cases. The first‐line treatments of metastatic castration‐resistant prostate cancer (mCRPC) include abiraterone acetate plus prednisone (AA/P), enzalutamide, radium 223 (Ra 223), docetaxel at 75 g/m^2^ every 3 weeks in association with prednisone or prednisolone, and sipuleucel‐T (see 16).^3^, ^4^, ^5^, ^6^, ^7^, ^8^ Despite the efficacy of these drugs, patients over time can be faced with serious toxicities, impaired quality of life, and cancer progression.^9^ Therefore, with a median overall survival of 2 years, mCRPC patients have a poor prognosis.^10^ All this information underlines the need to develop new therapeutic approaches with lower toxicity in order to improve mCRPC survival and quality of life. In this context, the use of complementary medicine against cancer could help to reduce toxicity. Furthermore, the combination of chemotherapy with noncytotoxic agents might provide a better outcome and response to chemotherapy with reduced toxicities.

Curcumin (diferuloylmethane) is a yellow coloring agent found in the roots of turmeric (*Curcuma longa*). It is used as food coloring and also in traditional medicine as an antiseptic and anti‐inflammatory agent.^11^, ^12^ Several studies have been conducted and have demonstrated that curcumin has many antineoplastic properties such as antiproliferative, antiangiogenic, and anti‐invasive effects, making it a potential treatment against cancer.^13^, ^14^, ^15^, ^16^ Furthermore, curcumin is a natural product known to be less toxic, to entail fewer side effects, and is therefore safe to use.^14^, ^17^ Besides its anticancer properties, some studies have also focused on the chemo‐potent role of curcumin, showing that it could be interesting to associate curcumin with chemotherapy in order to increase the action of chemotherapy on cancer cells.^18^, ^19^ Based on previous preclinical and clinical study results, a phase I study was conducted on advanced and metastatic breast cancer in Jean Perrin Comprehensive Cancer Center. It showed that the recommended dose of curcumin was 6000 mg/day for 7 consecutive days every 3 weeks in combination with a standard dose of docetaxel.^20^ These encouraging results allowed a phase II study to be conducted, which produced additional data on curcumin as a treatment for cancer, with a high response rate, good tolerance, and patient acceptability.^21^

This justified interest in conducting a randomized phase II trial to compare docetaxel plus curcumin versus docetaxel plus placebo. This study set out to evaluate the efficacy of docetaxel combined with curcumin, a polyphenolic derivative extracted from Curcuma longa root, as a first‐line treatment for mCRPC patients.

## 2 PATIENTS AND METHODS

### 2.1 Patients

Eligible patients were men (>18 years old) with histologically confirmed adenocarcinoma of prostate cancer, documented castration resistance, and at metastatic stage, defined by objective progression with at least one measurable lesion according to the RECIST 1.1 criteria and/or a rise in prostate‐specific antigen (PSA) level according to the PCWG2 criteria. At baseline, patients were to have an Eastern Cooperative Oncology Group (ECOG) performance status (PS) ≤2 with a life expectancy of at least 3 months and adequate functioning of major organs: bilirubin ≤ upper normal limit (UNL), AST and ALT ≤2 × UNL, alkaline phosphatase ≤2.5 × UNL or <10 × UNL in patients with bone metastasis without liver metastasis, serum creatinine <140 µmol/L, neutrophils ≥2 × 10^9^/l, platelets >100 × 10^9^/L, and haemoglobin ≥10 g/dl. Previous chemotherapy (except Estracyt), cerebral metastases, and concurrent severe or uncontrolled diseases were the exclusion criteria. A total of 100 patients were expected to participate, and an interim analysis was scheduled after the inclusion of 50 patients with outcomes at 6‐month post‐enrolment.

### 2.2 Study design

This was a randomized, double‐blind, phase II clinical trial conducted in three French Comprehensive Cancer Centers. This study was approved by the CPP Sud Est VI Ethics Committee (15/07/2013) and the national review board (Agence National de Sécurité des Médicaments et des produits de santé) (11/10/2013). The study was registered on ClinicalTrials.gov (NCT 02095717).

A total of 50 patients were included from June 2014 to July 2016, and all patients provided written informed consent prior to study enrolment. At baseline, pretreatment data were collected including medical history, previous treatments, and a physical examination with details about weight, height, body surface, and PS. A complete biology exploration was performed at baseline and before each chemotherapy cycle. Quality of life was evaluated using the self‐administered Quality of Life Questionnaire‐Core 30 (QLQ‐C30) and the Quality of Life Questionnaire‐Prostate 25 (QLQ‐PR25) at baseline and at the end of the treatment (Data S1). At each visit, any adverse events and concomitant medication details were recorded. Patient compliance was also checked via a notebook where patients indicated whether they had taken the capsules or not.

### 2.3 Treatment

Chemotherapy with docetaxel at 75 mg/m^2^ was administrated on Day 1 every 3 weeks for 6 cycles in association with continuous prednisone or prednisolone at 5 mg twice a day. Curcumin was formulated in 500‐mg capsules. Each patient received 6 g of curcumin or placebo per day (four capsules in the morning, four capsules at lunchtime, and four capsules in the evening) for 7 consecutive days (from Day −4 to Day +2 with chemotherapy at Day 0) every 3 weeks.

### 2.4 Study endpoints

The primary endpoint of the study was to evaluate the therapeutic benefit of docetaxel plus curcumin in comparison with docetaxel plus placebo, by calculating the time to progression (TTP) from inclusion to the first objective progression of the disease in the modified ITT population. Progression was defined as PSA progression with an increase of ≥25% and an absolute increase of 2 ng/ml (PSA level was to increase twice consecutively with a minimum interval of 2 weeks) (1) and/or a documented increase in target lesion(s) of at least 20% or the appearance of 1 or several new lesions according to the RECIST 1.1 criteria (2), and/or documentation of at least two new bone lesions on a bone scan (3).

The secondary endpoints were compliance, overall survival, PSA response, safety, assessment of curcumin absorption, and quality of life.

### 2.5 Response evaluation

Response evaluation was assessed by serum PSA level measures or tumor evaluation. PSA was measured every 3 weeks, and stability, response, and progression were defined according to the Prostate Cancer Clinical Trial Working Group 2 (PCWG2) criteria.^22^ Tumor evaluation was conducted at baseline and every 3 cycles during treatment and according to RECIST 1.1 criteria: complete response, partial response, stable disease, or progressive disease.

### 2.6 Curcumin titration

Curcumin absorption was evaluated by selecting six patients with good compliance in both arms. Nonhydrolyzed and hydrolyzed forms were titrated in blood samples taken at inclusion, Cycles 1, 3, and 6. The extraction procedure was based on the method previously validated by Heath et al., and curcumin titration was performed by liquid chromatography and mass spectrometry (Data S2).^23^

### 2.7 Statistical analysis

The analysis was primarily descriptive. Categorical parameters were described using the number of patients by class and the corresponding proportion. Quantitative values were presented with medians and ranges unless stated otherwise (means ± standard deviation). Student's *t* test was used to compare a numerical parameter between treatment groups if distributions were Gaussian and homoscedastic. Else, Kruskal–Wallis *H* test was preferred. For categorical parameters, Chi^2^‐test was used, and if class sizes were too small, Fisher's exact test was calculated. Survival curves were drawn using Kaplan–Meier's method, and Log‐rank test was computed to evaluate the difference between two curves. Difference on main objective was considered statistically significant at *p* < 0.03 for this interim analysis. However, standard *p* value was used for other secondary objectives. The efficacy analysis was conducted on the mITT population while the safety analysis was performed on the overall population, excluding the few patients who had not beginning the treatment. Database management and statistical calculation were performed using structural equation modeling (SEM) software.^24^

## 3 RESULTS

### 3.1 Patient characteristics

**FIGURE 1:**
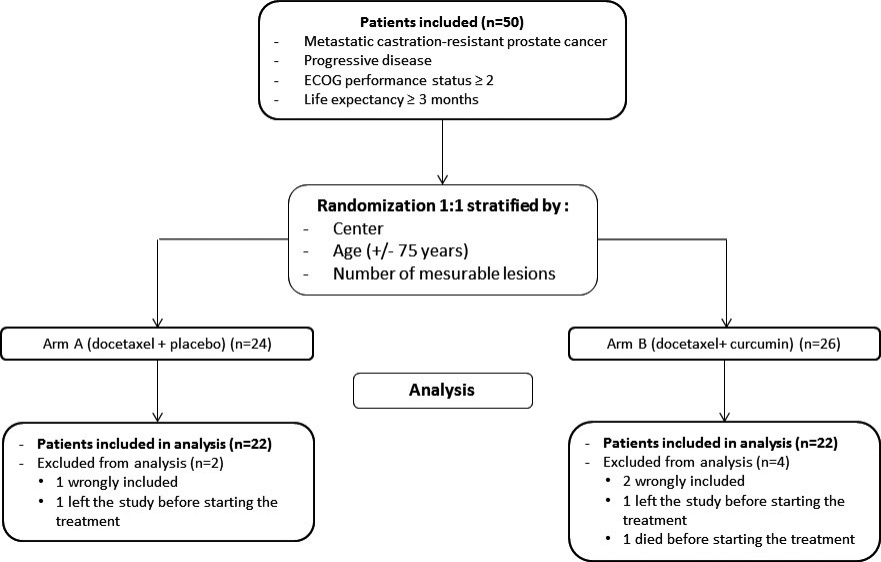
Flowchart of participants: A total of 50 patients were enrolled according to the inclusion criteria. (A) 1:1 randomization were done with stratification by center, age (+/− 75 years) and mesurable lesions; 24 patients were assigned to the arm A (docetaxel + placebo) and 26 patients to the arm B (docetaxel + curcumin). The interim analysis was conducted in the modified ITT population, that is, patients who were given at least one dose of docetaxel + curcumin/placebo. Among them, six patients (two in the arm A and four in the arm B) were excluded from the analysis: Three patients were wrongly included and did not meet the inclusion criteria; three other patients died or left the study before starting the treatment

A total of 50 patients were enrolled from June 2014 to July 2016 in this study, and the interim analysis was conducted in the modified ITT population, that is, patients who were given at least one dose of docetaxel + curcumin/placebo. Among them, six patients were excluded from the analysis: Three patients were wrongly included and did not meet the inclusion criteria, three other patients died or left the study before starting the treatment (Figure 1). Patients’ baseline characteristics are summarized per treatment arm in Table 1; 22 patients were analyzed in each arm: arm A (docetaxel + placebo) and arm B (docetaxel + curcumin). The median age was 69 and 70 years, respectively, for patients in arm A and arm B. The ECOG performance status was 1 or 2 for most patients in both arms. Concerning the Gleason score, the type of progression, the PSA level at baseline, the number of metastases, and the sites of metastases, no differences were found between the two arms. Patient characteristics were well balanced with an effective randomization process ensuring the comparison of patients in the two arms.

**TABLE 1:** Patient characteristics

	A	B	*p* value
Characteristics	*N*	*N*	
No. patients	22	22	—
Median age at enrolment (years) [range]	69 [60–80]	70 [44–87]	0.73
≥75 years	5	7	0.5
Gleason score	—	—	0.77
≤6	1	2	—
7	13	9	—
≥8	7	10	—
Not available	1	1	—
ECOG performance status	—	—	0.98
0	0	0	—
1	13	12	—
2	9	9	—
Unknown	0	1	—
Type of progression at baseline	—	—	—
At least one targeted lesion	10	12	0.77
PSA	22	19	0.23
Median PSA value at baseline (ng/ml) [range]	119 [7.0–1882]	50.6 [1.7–1587]	0.16
Previous surgery	21	21	0.47
Previous radiotherapy	15	14	0.75
Previous hormonotherapy	22	22	0.81
No. of sites of metastatic disease	—	—	1
1	15	14	—
2	2	3	—
3	3	2	—
Sites of metastases	—	—	0.88
Bone	17	18	—
Lymph node	6	4	—
Lung	3	3	—
Liver	2	1	—

Abbreviations: ECOG, Eastern Cooperative Oncology Group; PSA, prostate‐specific antigen.

### 3.2 Compliance

Compliance according to pharmacy statistics was 95.55% and 96.38%, respectively, for the placebo and curcumin arms. No difference was noticed between the two groups (*p* = 0.68) showing that the two groups of patients were comparable regarding their treatment fulfilment.

### 3.3 Progression‐free survival

**FIGURE 2:**
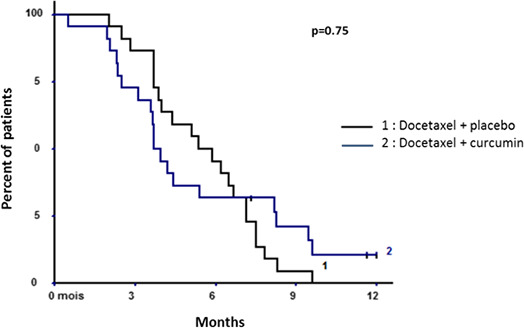
Progression‐free survival in the modified intention‐to‐treat population

The median progression free‐survival (PFS) in the modified ITT (mITT) population was 4.4 months. At 6 months, 38.6% of patients were alive without any documented progression. No difference was shown for PFS between the two arms (median PFS of 5.3 months in the docetaxel + placebo group vs. 3.7 months in the docetaxel + curcumin group; *p* = 0.75) (Figure 2). At 6 months, the percentage of PFS was 45.5% in the placebo arm versus 31.8% in the experimental arm; this difference was not significant (*p* = 0.35). This study initially hypothesized a superiority of at least 25% in the treatment arm, but there was a 14% difference in favor of the placebo group. To determine whether the study could be continued, despite this unfavourable unbalance, the chances of having a 25% difference in favor of the experimental group if accrual is continued till the end of the study were analyzed, and the chances to end up with a positive outcome are only 2%.

### 3.4 Secondary endpoints

#### 3.4.1 Global survival

**FIGURE 3:**
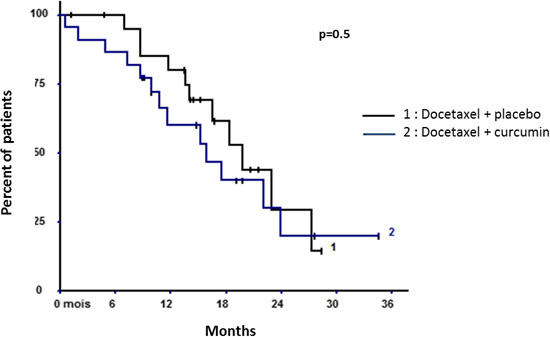
Overall survival in the modified intention‐to‐treat population

The median overall survival was 18.4 months in the modified ITT population. Comparing the two arms, the median overall survival was 19.8 months versus 15.8 months, respectively, for the placebo and curcumin groups (*p* = 0.50) (Figure 3). At 12 months, the survival rate was 80.0% versus 60.1%, respectively, for the placebo and curcumin groups (*p* = 0.17). At 24 months, the survival rate was 29.3% versus 20.0%, respectively, for the placebo and curcumin groups (*p* = 0.62).

#### 3.4.2 PSA response and evolution

**FIGURE 4:**
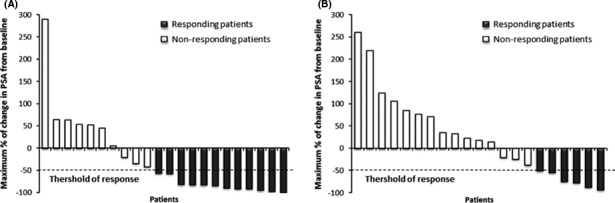
Waterfall plot showing the maximal percentage of change in prostate‐specific antigen (PSA) post‐therapy from baseline. The thershold of response is defined by a decrease of ≥50% during the treatment period. The last PSA value was taken for patient who discontinued the treatment before a minimum exposure of 12 weeks. (A) Waterfall plot of patients in the placebo group, *n* = 22. (B) Waterfall plot of patients in the curcumin group, *n* = 21 (one patient was excluded from the waterfall plot because no PSA value was available after baseline)

**FIGURE 5:**
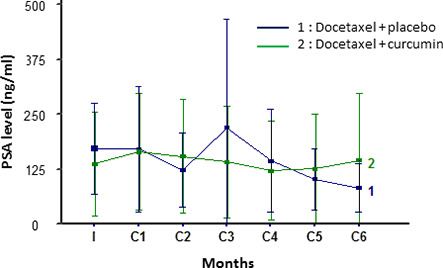
Prostate‐specific antigen (PSA) evolution in the modified intention‐to‐treat (mITT) population

PSA response was defined as a reduction in serum PSA level of at least 50% and has been summarized in Figure 5. Waterfall plot analysis revealed a PSA decline of ≥50% in 12 patients receiving docetaxel + placebo (Figure 4A) and in six patients receiving docetaxel + curcumin (Figure 4B).

Exploration of PSA evolution over the entire treatment period showed no difference between the two arms (*p* = 0.88) (Figure 5).

#### 3.4.3 Safety

Among the 44 patients, the most common toxicities were anemia, asthenia, diarrhea, and alopecia. Nothing relevant was noted between the two groups of patients, except less lymphopenia (*p* = 0.023) and less hypocalcemia (*p* = 0.021) in the experimental arm. The incidence of Grade 3–4 adverse events (AEs) is reported in Table 2, and no difference was highlighted between both treatment groups.

**TABLE 2:** Treatment related adverse event at Grade 3 or 4

Adverse event	Arm A: docetaxel +placebo	Arm B: docetaxel +curcumin
	Grade 3–4	Grade 3–4
Neutropenia	3	3
Leucopenia	2	1
Anemia	2	0
High phosphatase alkaline	2	0
Lymphopenia	1	1
Edema of the lower limbs	0	1

A total of 15 serious adverse events (SAEs) were notified, concerning 11 patients among the 50 patients included in the study. Among them, only two were attributed to chemotherapy with docetaxel and none of them to curcumin. Three patients died during the treatment period of the study, but none of these deaths were related to the study protocol.

#### 3.4.4 Curcumin absorption

**FIGURE 6:**
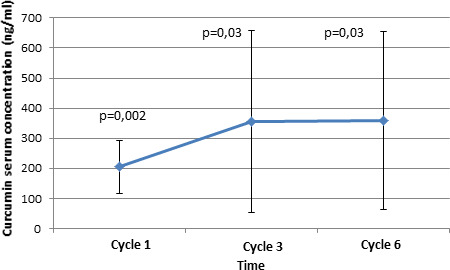
Curcumin serum concentration (*p* value = comparison of means to zero) (*n* = 6)

The nonhydrolyzed form of curcumin was not detectable (<2 ng/ml) in either arm. Concerning the hydrolyzed form, the values obtained in the placebo and curcumin arms were compared. In both groups, no curcumin was detected at inclusion. At Cycles 1, 3, and 6, all patients in the placebo arm had a hydrolyzed curcumin value under the limit of quantification (LOQ) so the value obtained in the curcumin arm was compared with 0. For Cycles 1, 3, and 6, the difference between the two arms was significant with *p* = 0.002, *p* = 0.03, and *p* = 0.03, respectively (Figure 6). In the analysis of patients who received curcumin, no cumulative effect of curcumin could be evidenced between Cycles 1–3 and Cycle 3–6 (*p* = 0.26 and *p* = 0.99), but interindividual variability was observed (*p* = 0.0065).

#### 3.4.5 Quality of life

Concerning quality of life, no differences were noted between the two arms (*p* = 0.49 and 0.47, respectively, for the QLQ‐C30 and the QLQ‐PR25) (Data S3a and b).

## 4 DISCUSSION

Several preclinical and clinical studies have been conducted and have shown the benefits of curcumin as an antineoplastic component and also as a chemopotent agent, making it a potential treatment against cancer.^11^, ^18^ These previous results led us to conduct research using curcumin as an agent associated with chemotherapy based on docetaxel. After a phase I study and a nonrandomized phase II trial on curcumin, this was the first randomized double blind study conducted in mCRPC.^20^, ^21^ Its aim was to see whether the association of curcumin with docetaxel provided any benefit on patient prognosis and on PFS in particular.

The primary endpoint of the study was a PFS increase of at least 25% in favor of the curcumin group. Despite a well‐balanced population in the two arms and a good compliance rate, the results from the interim analysis showed a clear lack of efficacy of adding curcumin to the current mCRPC treatment. Our result showed a better PFS in the placebo group but without reaching significance. The results from the interim analysis did not justify continuing the study. This result could be an effect of the small sample size, which should not be neglected. Indeed, if the study had been pursued until a total of 100 patients, the chances of concluding in favor of curcumin would have been very small. Because of this, as declared in the protocol, the study was stopped after the interim analysis on the grounds of futility. We conclude that the addition of curcumin to docetaxel in mCRPC has no effect on PFS. Moreover, although the follow‐up time was short, the overall survival rate was examined and was not found to be conclusive either. Alongside, the results showed no effect of curcumin on PSA response or quality of life. On the other hand, the safety analysis did not show any harmful effect of curcumin, with a good tolerance among patients, which confirms the literature data.^14^

The association of curcumin with docetaxel does not seem to potentiate the chemotherapy effect on cancer cells, but at the same time, no side effects of curcumin were noted. Our negative results could be attributed to the poor bio‐availability of curcumin.^25^ Indeed, many studies have focused on the unstable character and very low absorption of curcumin.^26^, ^27^ To strengthen our data and to know whether curcumin absorption and metabolism were satisfactory in our study, serum curcumin levels were assessed in 12 patients (six per arm). Naturally, curcumin and its derivatives were detected only in patients in the experimental arm. No cumulative effect of curcumin was shown, but interindividual variability was observed. These results underline that curcumin is indeed absorbed and bioavailable, but this study does not provide sufficient information on the optimal dose of curcumin that could be efficient. The interindividual variability evidences a difference in curcumin metabolism: Perhaps the dose of curcumin should be personalized to increase its effect. Possibly a better absorption of curcumin would have provided better results. In fact, as shown and recommended by some other studies, curcumin absorption could be enhanced by more efficacious associations or a better formulation. As an example, curcumin associated with piperine, with phosphatidyl choline, or nanoparticles and nanocrystal formulations of curcumin seem to have better bio‐availability.^25^, ^26^, ^27^, ^28^, ^29^, ^30^

Our study has some limitations including the small sample size as a result of the early discontinuation at the interim analyses, and the titration of curcumin could have been performed for all patients included (50) and not only a few patients (12) in order to strengthen the results on curcumin bio‐availability. On the other hand, this study was designed with a robust methodology (randomized, double‐blind, placebo‐controlled, and multicentre), and it is the first one to explore the efficacy of curcumin in mCRPC treated with docetaxel, with the assessment of serum curcumin levels.

According to our previous studies on curcumin and the literature data, curcumin associated with chemotherapy and specifically with docetaxel seemed to be a promising lead in cancer treatment.^19^, ^31^, ^32^ The results of this phase II study lead us to conclude that in mCRPC patients, oral curcumin combined with docetaxel at the studied posology (6g/d for 7 days) does not improve either progression‐free survival or overall survival. We could not neglect that the small sample size could be a reason of absence of difference between the two arms. As a result of this study, curcumin cannot be recommended in association with chemotherapy in mCRPC, but further studies with more patients are required to examine whether curcumin could be of interest in other cancer treatments and to see if improved formulations of curcumin could increase its efficacy.

## Supporting information

Data S1‐S3

## Data Availability

The data that support the findings of this study are available from the corresponding author upon reasonable request.
