# Supplementary material for: Multicenter randomized phase II study comparing docetaxel plus curcumin versus docetaxel plus placebo in first‐line treatment of metastatic castration‐resistant prostate cancer
Source: Cancer Med. 2021 Mar 5;10(7):2332–40. doi: 10.1002/cam4.3806 (PMC7982628; doi:10.1002/cam4.3806)
Supplement: Supplementary file 1 — Data S1‐S3 [file CAM4-10-2332-s001.docx]

**Supporting information**

**SuppInfo.1: Study design**

At inclusion, pre-treatment data was collected including medical history, previous treatments, physical examination (weight, height, body surface, and PS), scans (CT scan and bone scan), and a complete biology exploration. Quality of life was evaluated using the self-administered Quality of Life Questionnaire-Core 30 (QLQ-C30) and the Quality of Life Questionnaire-Prostate 25 (QLQ-PR25) at baseline and at the end of the treatment. CT scan and bone scan were repeated after cycle 3 and cycle 6 and every 3 months until progression. After the 6 cycles of treatment, patients were in follow-up until progression.


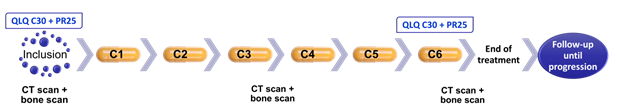


Abreviations: C: chemotherapy cycle, CT scan: computerized tomography, QLQ-C30: Quality of Life Questionnaire-Core 30, QLQ-PR25: Quality of Life Questionnaire-Prostate 25

**SuppInfo.2: Details concerning the extraction procedure and curcumin titration**

For plasma samples: The extraction procedure was based on the method previously validated by Heath *et al*.^22^. The analysis samples were prepared by placing 20µL of IS (β-estradiol-17 acetate at 5g/L) into 500µL of biological sample. Sample solutions were extracted with 5mL of ethyl acetate / methanol (95/5; v/v). The upper organic layer was collected, evaporated under a stream of nitrogen at room temperature and the extracts were then reconstituted in methanol (100µL) before HPLC analysis.

Enzymatic hydrolysis of curcumin conjugate: Samples were also assayed for curcumin and its conjugates after incubating the plasma samples with the enzymes β-glucuronidase and sulfatase (Helix Pomatia HP-2).

Liquid chromatography and mass spectrometry: Liquid chromatography and mass detection methods were the same for free and total curcumin and metabolite sample analyses. On-line chromatography purification was carried out using a turbulent flow column (cyclone 0.5 x 50 mm) at 1.5mL/min for 30s (transcend TLX1, Thermo Fisher Scientific, San Jose, CA, USA). The elution step used 200µL of acetonitrile/water (0.1% formic acid) (80/20; v/v). Chromatographic separation was carried out using a reverse-phase liquid at 30°C using a Hypersil GOLD column (50 x 2.1 mm, 1.9µm) (Thermo Fisher Scientific, San Jose, CA, USA). A gradient system with the mobile phase consisting of solvent A (0.1%; v/v; formic acid in water) and solvent B (0.1%; v/v; formic acid in acetonitrile) at a flow rate of 500µL/min. Running time was set at 11.42min. The auto sampler was kept at 4°C.

On-line LC-MS/MS analyses were performed on a 4000QTrap triple quadripole linear trap mass spectrometer equipped with a turbo ionspray source operated in electrospray mode (ABSciex, Fester City, CA, USA). MS experiments were performed with a multiple reaction monitoring (MRM) condition in negative mode. The MRM transition of m/z 367.03 🡺 133.80 for curcumin, m/z 337.05 🡺 216.80 for DMC, m/z 306.97 🡺 186.70 for BDMC, and m/z 313.05 🡺 252.90 for β-estradiol-17acetate were simultaneously monitored. The concentration of curcumin and metabolites in biological samples, were determined by the ratio of their area to that of the IS, using a weighted quadratic fit. The lower limit of quantification (LLOQ) for each compound was 1µg/L and the upper limit of quantification (ULOQ) was 100µg/L in plasma.

**SuppInfo.3: Data concerning quality of life**


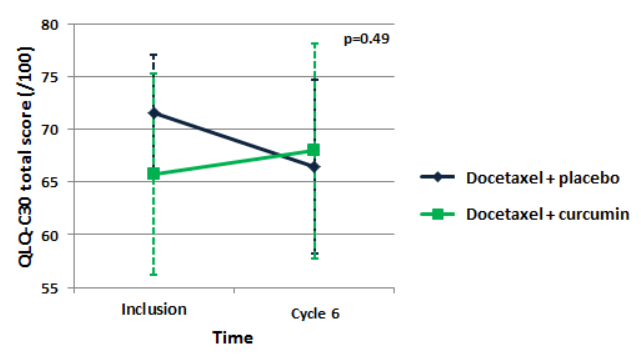


1. **QLQ-C30 total score variation from inclusion to cycle 6 of chemotherapy :** mean (± 95% confidence intervals).


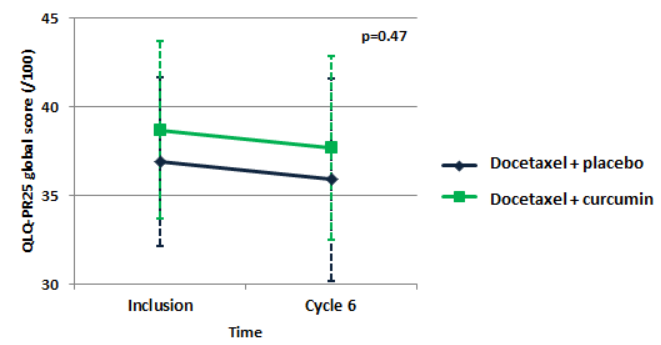


1. **QLQ-PR25 global score variation from inclusion to cycle 6 of chemotherapy** mean (± 95% confidence intervals).
